# Effect of testing procedures on gait speed measurement: A systematic review

**DOI:** 10.1371/journal.pone.0234200

**Published:** 2020-06-01

**Authors:** Anna K. Stuck, Madeleine Bachmann, Pia Füllemann, Karen R. Josephson, Andreas E. Stuck

**Affiliations:** 1 Department of Geriatrics, Inselspital, Bern University Hospital, University of Bern, Bern, Switzerland; 2 Geriatric Research Education & Clinical Center, Veterans Administration Greater Los Angeles Healthcare System, Los Angeles, California, United States of America; Cardiff University, UNITED KINGDOM

## Abstract

**Background:**

Although gait speed is a widely used measure in older people, testing methods are highly variable. We conducted a systematic review to investigate the influence of testing procedures on resulting gait speed.

**Methods:**

We followed the PRISMA checklist for this systematic review. Two independent reviewers screened Pubmed and Embase for publications on pairwise comparisons of testing procedures of usual gait speed. Descriptives were abstracted from the included publications using a predefined extraction tool by two independent reviewers. We defined the cut-off for the minimal clinically imporant diffence in gait speed as 0.1 m/sec.

**Results:**

Of a total of 2109 records identified for screening, 29 reports on 53 pairwise comparisons were analyzed. The median (range) difference in gait speed for dynamic versus static start was 0.06 (-0.02 to 0.35) m/sec (14 reports); for longer versus shorter test distance 0.04 (-0.05 to 0.23) m/sec (14 reports); for automatic versus manual timing 0.00 (-0.05 to 0.07) m/sec (12 reports), for hard versus soft surfaces -0.11 (-0.18 to 0.08) m/sec (six reports), and electronic walkways versus usual walk test 0.04 (-0.08 to 0.14) m/sec (seven reports), respectively. No report compared the effect of finishing procedures.

**Conclusions:**

The type of starting procedure, the length of the test distance, and the surface of the walkway may have a clinically relevant impact on measured gait speed. Manual timing resulted in statistically significant differences of measured gait speed as compared to automatic timing, but was below the level of clinical importance. These results emphasize that it is key to use a strictly standardized method for obtaining a reliable and valid measurement of gait speed.

## Introduction

Gait speed is recognized as a valid and reliable predictor and outcome measure of multiple aspects of physical function in older people and patients. In fact, gait speed is considered the sixth vital sign for older patients [1]. Moreover, gait speed is recognized internationally and across disciplines (e.g. geriatrics [2], orthogeriatrics [3], neurology [4], nephrology [5], cardiology [6], women’s health [7]) as an essential component in the assessment of older patients.

Evidence demonstrates that gait speed is associated with functional impairment, cognitive decline [8], disability [9] and mortality [10]. In some conditions, gait speed is used as part of the diagnostic criteria and in determining the need for medication and therapy. For example, the ICD diagnosis [11] for severe sarcopenia requires a gait speed slower than 0.8 m/sec.

Although numerous papers recommended methods for the measurement of usual gait speed [12, 13], there is currently no general agreement on a detailed testing protocol defining all known methodological components for measuring gait speed. Several recent reviews actually confirm that the methods used for measuring gait speed at usual speed differ between studies, including the distance walked, starting and deceleration procedures, timing, and type of testing surface [13–15]. Two reviews pooled data from studies measuring gait speed and found that these differences in testing methods did not affect the resulting gait speed [14, 15]. However, this approach is not sensitive for detecting the potential impact of testing procedures on gait speed results, because patient and testing confounders cannot be adequately controlled for, and confounders are therefore likely to mask the real effect of testing procedures on resulting gait speed. Studies comparing different approaches of testing procedures (pairwise comparisons) within the same population are needed to determine the effect of testing procedures on resulting gait speed. Over the past years, multiple studies using this approach have been published with variable results, but there is to our knowledge no systematic review on the findings of these studies. To address this research gap, we performed a systematic review of published research to investigate if different testing procedures have a clinically relevant impact on gait speed.

## Methods

### Searching and screening

We conducted a systematic search in Pubmed and Embase using a protocol based on the PRISMA statement for conducting and reporting systematic reviews [16]. No language or time restrictions (up to April 15 2020) were applied. We identified additional articles by searching cited references of relevant articles. The detailed search strategy is shown in the Supporting information.

We included original and published articles that compared two methods of measuring gait speed over a maximum distance of 10 meters and that reported quantitative data on gait speed in m/sec or the equivalent in adults. We excluded articles that assessed only one measurement method, reported results of the 6-minute walk test, compared different computerized electronic walkway systems, or only reported comparision of usual versus maximal gait speed. Non-English articles, letters, reviews, and editorials were also excluded. Titles and abstracts were screened by two independent reviewers, who then performed a full-text screening based on the eligibility criteria. Discrepancies were resolved by a third reviewer.

Publications that included pairwise comparisons between testing methods for usual gait speed were identified. If a study made a comparison in the same study population more than once (e.g. for reliability purposes), the results of the first comparison were extracted for the systematic analysis.

### Data extraction

Two reviewers independently extracted information using a standardized predefined data extraction tool. Additional methodological references and appendices included in the reviewed article were consulted as well. We extracted study and patient characteristics, descriptive data for each pairwise comparison and corresponding gait speed results. Two investigators independently extracted data, and disagreements were resolved by a third reviewer.

### Risk of bias

We assessed the methodological quality of included studies based on the Agency for Healthcare Research and Quality (AHRQ) methodology checklist. The checklist domains assess inclusion of: the source of information, inclusion and exclusion criteria, time period, consecutive recruitment, masking, quality assurance, controlling of confounders, handling of missing data, response rate and completeness of data. This 11-item checklist is considered applicable for cross-sectional studies [17]. Each item is scored “yes” (1 point), “no” (0 point) or “not applicable”. Two independent reviewers used the checklist to score each study and a total score was calculated. Final scores ranged from 0–11, where 11 points suggested “lowest risk of bias” and 0 corresponded to “highest risk of bias”. A difference of 2 or more points was considered discrepant and decided by consensus. From the AHRQ checklist, we calculated a percentage for the overall risk of bias for each study dividing the number of items with 0 points by the total number of applicable items (i.e. total number of 11 items minus the number of non-applicable items for each study).

### Analysis

For qualitative synthesis, stratification was performed by the type of pairwise comparison: 1) starting procedures (e.g. static (no acceleration) vs. dynamic start (distance for acceleration)), 2) distance procedures (e.g. 4 m vs. 10 m), 3) timing procedures (e.g. manual vs. automatic), 4) surface procedures (e.g. hard vs. soft ground), 5) walkway procedures (e.g. electronic walkway vs. overground surface) and 6) finishing protocols (deceleration vs. no deceleration).

In a summative approach, we combined results of included studies by calculating medians and ranges of absolute and relative differences of gait speed results and intraclass correlation coefficients, stratified by the type of pairwise comparison. Descriptive data are shown as numbers and percentages for categorical variables, and median and ranges for numerical variables. We considered a gait speed difference of 0.1 m/sec as clinically relevant, based on a systematic review on the minimal clinically important difference (MCID) of gait speed measurement [18].

## Results

### Study selection

**Fig 1:**
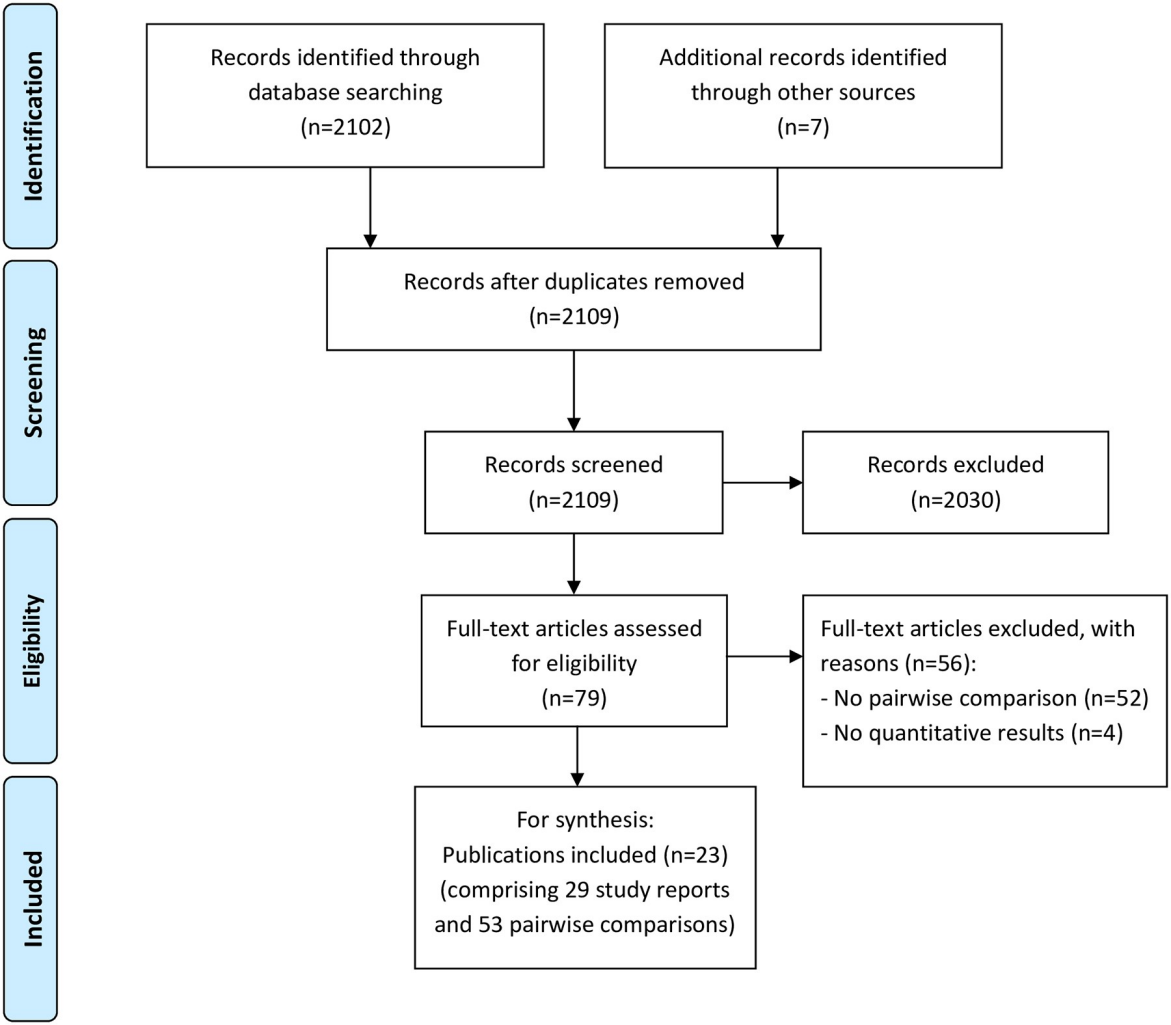
Flow chart.

A total of 2109 studies were identified through the search strategy (Fig 1). Of these, 2030 studies were excluded after screening the abstract, leaving 79 studies for full-text screening to assess eligibility. Overall, 23 publications comprising 29 study reports and 53 pairwise comparisons were included in the systematic synthesis.

### Study report characteristics

Table 1 describes descriptive characteristics of included study reports (n = 29). Overall, 47% of participants were female with a mean age of 62.9 years (SD 16.0). The majority of reports recruited study participants from community settings (n = 23), although three reports were conducted with rehabilitation patients [19–21], and one with hospitalized patients [22]. Two reports did not specify the setting [23, 24]. Thirteen reports (45%) specifically targeted patients suffering from an underlying disease (stroke n = 5 [19, 20, 25], Parkinson disease n = 3 [24, 26, 27], and pulmonary disease n = 2 [21, 28]). The majority of study reports were observational cohort studies (n = 27), but two reports used a case-control design [29]. Overall, only four were multicenter studies [20, 30–32]. Thirteen reports investigated intertest (i.e. intra-rater) reliabilty and/ or agreement [19, 25, 27, 28, 31–36], while only two reports analyzed interobserver (.i.e. inter-rater) reliability and/or agreement [21, 37].

**Table 1:** Characteristics of included study reports (n = 29).

Author and year of publication; country	Mean age, (years)	Proportion female, (%)	Study setting	Study population characteristics	Reliability and/or agreement measures	Risk of bias, (%)	Categories of testing procedures evaluated
Amatachaya 2019a; Thailand[38]	37	73	Community	Young healthy	No	87.5	Starting, distance
Amatachaya 2019b; Thailand[38]	78	52	Community	Older healthy	No	87.5	Starting, distance
Amatachaya 2019c; Thailand[38]	59	19	Community	Spinal cord injury patients	No	87.5	Starting, distance
Barry 2018; USA[33]	81	66	Community	Individuals who visited geriatric clinic and could walk independently	Yes, inter-test	22.2	Timing
Bisca 2018; Brazil[21]	69	43	Rehabilitation	Patients with COPD	Yes, inter-observer	50.0	Timing
Bohannon 2008; USA[30]	N.r.	49	Community	Noninstitutionalized individuals	No	20.0	Distance
Bryant 2013; USA[26]	69	30	Community	Patients with idiopathic Parkinson disease off medication	No	77.8	Surface walkway
Bryant 2015; USA[24]	66	0	N.r.	Parkinson patients	No	100.0	Walkway
Cleland 2019; USA[23]	59	31	N.r.	Patients with chronic hemiparesis	No	88.9	Walkway
Johnson 2020a; USA[35]	20	42	Community	Young healthy	Yes, inter-test	66.7	Starting, distance
Johnson 2020a; USA[35]	25	70	Community	Young healthy	Yes, inter-test	66.7	Starting, distance
Karpman 2014; USA[28]	66	40	Community	Patients with clinically stable COPD	Yes, inter-test	66.7	Distance, timing
Kim 2019; South Corea[31]	76	53	Community	Non-disabled community-dwelling individuals aged 70 years and older	Yes, inter-test	33.3	Starting, timing
Lindholm 2018; Sweden[27]	68	45	Community	Outpatients with Parkinson disease	Yes, inter-test	36.4	Starting
Lyons 2015; USA[32]	76	64	Community	Care-givers and non-caregivers aged 60+ years	Yes, inter-test	33.3	Distance
Ng 2012; China[20]	59	20	Rehabilitation	Patients with chronic stroke	No	66.7	Distance
Ng 2013; China[39]	60	60	Community	Healthy older adults 50+	No	77.8	Distance
Oh 2019; South Corea[37]	75	63	Community	Healthy older adults aged ≥ 65 years	Yes, inter-observer	66.7	Starting, timing
Pasma 2014; Netherlands[40]	82	65	Community	Community-dwelling elderly referred to a geriatric outpatient clinic	No	50.0	Distance
Peters 2013; USA[34]	84	74	Community	Healthy older adults aged 65+	Yes, inter-test	30.0	Distance, timing
Peters 2014a; USA[25]	63	42	Community	Patients with chronic unilateral stroke: Community ambulators subgroup	Yes, inter-test	44.4	Walkway
Peters 2014b; USA[25]	65	17	Community	Patients with chronic unilateral stroke: Limited community ambulators subgroup	Yes, inter-test	44.4	Walkway
Peters 2014c; USA[25]	60	33	Community	Patients with chronic unilateral stroke: Household ambulators subgroup	Yes, inter-test	44.4	Walkway
Promkeaw 2019a; Thailand[29]	50	23	Community	Ambulatory individuals with incomplete spinal cord injury	No	75.0	Surface
Promkeaw 2019b; Thailand[29]	55	43	Community	Ambulatory able-bodied individuals	No	75.0	Surface
Stephens 1999; Australia[19]	61.5	50	Inpatient rehabilitation	Stroke patients	Yes, inter-test	44.4	Surface
Sustakoski 2014; USA[41]	77	73	Community	Community-dwelling older adults age 65+ years	No	55.6	Starting, timing, walkway
Warden 2019; USA[36]	45	71	Community	Participants who could ambulate ≥10m	Yes, inter-test	55.6	Starting, timing
Willmott 1986; U.K.[22]	76	N.r.	Hospital	Elderly hospitalized patients	No	88.9	Surface

Abbreviations: COPD, chronic obstructive pulmonary disease; N.r., not reported.

Most reports evaluated the impact of one testing procedure (e.g. timing) on the outcome of gait speed. However, some reports evaluated pairwise comparisons of different test procedures in the same study population (e.g. one pairwise comparison on timing methods and another pairwise comparison on distance).

Morever, Table 1 depicts the risk of bias for each report. Overall, we found the largest median risk of bias in surface test procedures (75.0%) and the lowest median risk of bias in timing test procedures (41.7%) (Table 2). Overall, 17 of 29 reports had a high risk of bias of >50% and 12 reports had a low risk of bias of less than 50%.

**Table 2:** Impact of testing procedures on gait speed results of pairwise comparisons (n = 53).

Test procedure (method 1 versus method 2)	Number of pairwise comparisons, (n)	Median of mean differences in gait speed method 1 vs. 2, (m/sec) median (range) ^e)^	Intraclass correlation coefficient, median (range)	Overall risk of bias, (%) median (range)
**Starting test procedures (dynamic vs. static)**	14	0.06 (-0.02 to 0.35)	0.98 ^a)^	66.7 (33.3 to 87.5)
**Distance test procedures (longer vs. shorter distance)**	14	0.04 (-0.05 to 0.23)	0.80 (0.79 to 0.93) ^b)^	66.7 (20.0 to 87.5)
**Timing test procedures (automatic vs. manual)**	12	0.00 (-0.05 to 0.07)	0.99 (0.91 to 1.00) ^c)^	41.7 (22.2 to 66.7)
**Surface test procedures (soft vs. hard surface)**	6	-0.11 (-0.18 to 0.08)	N.a.	75.0 (44.4 to 88.9)
**Walkway test procedures (electronic walkway vs. usual walk test)**	7	0.04 (-0.08 to 0.14)	0.81 (0.49 to 0.96)^d)^	55.6 (44.4 to 100.0)
**Finishing test procedures (deceleration vs. no deceleration distance)**	0	N.a.	N.a.	N.a.

Abbreviations: vs, versus; N.a.; not available.

^a)^ n (number of pairwise comparisons) = 1

^b)^ n (number of pairwise comparisons) = 3

^c)^ n (number of pairwise comparisons) = 5

^d)^ n (number of pairwise comparisons) = 6

^e)^ Calculated as the absoulute difference of gait speed of method 1 (e.g. dynamic) minus method 2 (e.g. static). A positive value indicates that method 1 (e.g., dynamic) resulted in a faster gait speed compared to method 2 (e.g. static).

### Characteristics of pairwise comparisons

Overall, 29 study reports had information on 53 pairwise comparisons. Among these pairwise comparisons, 14 (26%) compared different approaches for the starting procedure including data of 3814 participants. Pairwise comparisons on starting procedures compared a static start (0 m acceleration distance) with a dynamic start (range: 0.5 to 3 m) (S1 Table). Fourteen (26%) pairwise comparisons compared the impact of the length of the testing distance on resulting gait speed including data of 2695 participants. Thereby, a longer distance (range: 6.1 to 10 m) was compared with a shorter distance (range: 2.4 to 5 m) (S2 Table). Twelve (23%) pairwise comparisons compared automatic timing system (sensor-based) with a manual timing (e.g. stopwatch) including data of 3797 participants (S3 Table). Six (11%) pairwise comparisons compared surface (soft vs. hard surface) test procedures including data of 222 participants (S4 Table). Seven (13%) compared walkway procedures with usual surface including data of 182 participants (S5 Table).

None of the identified pairwise comparisons evaluated only the impact of finishing procedures (Table 2).

Pairwise comparisons were heterogenous related to the methodologic approach used, with important differences on the characteristics of the study, and elements of the measurement protocols, that are summarized in S1–S5 Tables.

### Effect of testing procedures on gait speed

Table 2 summarizes the effect of the different testing procedures on resulting gait speed. In the Supplementary information, S6 to S10 Tables depict the mean values of the difference in gait speed observed in each pairwise comparison. These Tables also demonstrates that most studies reported an exact p-value for this mean difference, but 95% confidence intervals of the mean difference were often not reported.

Starting test procedures: In studies with a static start, participants are instructed to walk at their usual pace and time is measured immediately upon initiation of walking and includes the acceleration phase in the overal time measure. In contrast, for a dynamic start, timing usually begins 2 m after walking is initiated, so the acceleration phase is not included in the overall time. Among the 14 observations comparing the effect of starting test procedures, the median of mean differences in observed gait speed was 0.06 m/sec, with a higher gait speed for dynamic start as compared to static start. The MCID of 0.1 m/sec was exceeded in 4 of the 14 pairwise comparisons (S6 Table). Five comparisons also found a statistically significant higher mean gait speed for dynamic start, but below the MCID of 0.1 m/sec. In three comparisons the difference was not statistically significant, probably related to small sample sizes. Finally, as an exception, one study [27] found a statistically significant lower gait speed (-0.02 m/sec) for dynamic start as compared to static start in a sample of patients with mild Parkinson disease. The 95% confidence interval of this mean difference did not exceed the MCID (–0.008 to -0.026 m/sec). Of the 14 pairwise comparisons, only one reported an intraclass correlation coefficient (ICC 0.98) (Lindholm [27]).

For distance test procedures we observed an overall median absolute difference of 0.04 m/sec with a trend towards higher gait speed measured over the longer distance as compared to the shorter distance. Test distances varied between 2.4 and 10 m in these comparisons. There were 5/14 pairwise comparisons demonstrating that the difference in measured gait speed exceeded the MCID, all reporting higher gait speed results for the longer distance compared with the shorter distance (S7 Table). Two studies found statistically significant differences in measured gait speed below the MCID, but these studies did not report confidence intervals. The remaining 7 observations found no statistically significant differences. Three pairwise comparisons reported an intraclass correlation coefficient (median 0.80 (range 0.79 to 0.93).

For timing test procedures, the median absolute difference was 0.00 m/sec. Overall, MCID was exceeded in none (0%) of the pairwise comparisons on timing procedures (S8 Table). Three pairwise comparisons reported a statistically significant difference ([31], [31], [37]) in resulting gait speed in both directions, but 95% confidence intervals of mean differences did not exceed MCID. The remaining comparisons did not find statistically significant differences in measured gait speed for automatic versus manual timing. Five of the 12 pairwise comparisons on timing test procedures reported intraclass correlation coefficients with a median of 0.99 (range 0.91 to 1.00).

The absolute median difference in gait speed was -0.11 m/sec for surface test procedures. Most comparisons found slower gait speed for soft surfaces (e.g. carpet or grass) compared to hard surfaces. Overall, MCID is exceeded in 4/6 of surface test procedures (S9 Table). These studies hypothesized that soft or uneven surfaces may cause the test person to reduce usual walking speed. Interestingly, one study (Wilmott[22]) found a statistically signifcant correlation in the other direction, which was below the MCID. In this study, geriatric inpatients exhibited a slower gait speed (mean difference 0.08 m/sec) on a vinyl floor as compared to a carpeted floor. The authors suggested that patients overall felt unsafe while walking. Consequently, they may have been more cautious on a hard surface than on the softer carpeted surface. No intraclass correlations were reported for surface test procedures.

For walkway test procedures, the absolute median difference in gait speed was 0.04 m/sec. Overall, all pairwise comparisons were statistically significant, but in both directions. Five pairwise comparisons found higher gait speed results and two found slower results when electronic walkways were compared with usual surfaces. MCID was only exceeded in one pairwise comparison (Peters 2014 [25]) showing higher gait speed results for the electronic walkway than the usual walk test, but without reporting a 95% confidence interval. Moreover, the 95% confidence interval of the mean difference was exceeded in two pairwise comparisons: Cleland et al. [23] found lower gait speed results for the electronic walkway than usual surface of -0.08 m/sec (95% CI -0.05 to -0.1 m/sec), and Sustakoski et al. [41] found higher gait speed results on the electronic walkay compared with the usual surface of 0.07 m/sec (0.04 to 0.1 m/sec). Of note, Peters et al. (2014) [25] investigated three study subpopulations when comparing a usual walk test (3m) with a GAITrite walkway and found results in both directions. Among participants with good mobility, gait speed was significantly faster on the GAITRite walkway. In contrast, participants with impaired mobility had significantly slower gait speeds on the GAITRite walkway. Six of seven pairwise comparisons described an intraclass correlation coefficient with a median of 0.81 (range 0.49 to 0.96).

We did not find any studies that reported pairwise comparisons of finishing test procedures. However, with the exception of one pairwise comparison on starting test procedures (Lindholm[27]), all starting test procedures also had a distance for decleration upon dynamic starting protocols, but no distance for deceleration was provided on static starting protocols. Overall, most pairwise comparisons reported whether participants were told to stop at the finishing line (thereby including the deceleration phase in the timing of gait speed), or to stop after the finishing line (excluding deceleration from the gait speed measure). Distances of 1.5 m (24) and 2 m (16, 34) were reported for deceleration after the finishing line.

## Discussion

This systematic review found that five individual components of gait testing protocols may have a clinically relevant impact on gait speed results. Although we did not find studies comparing finishing procedures, this component likely affects the measurement of gait speed as well. To our knowledge this is the first systematic review summarizing studies that compared the impact of testing procedures on resulting gait speed based on pairwise comparisons. We found a considerable number of pairwise comparisons (n = 53) for this systematic analysis.

Of note, we report the median and range of mean differences as reported by the invidual original study reports, and did not pool the data using a meta-analytic approach due to the important methodological heterogeneity between the studies. First, as shown in S1 to S5 Tables, individual studies were based on different patient populations, with regard to age, health status and level of underlying gait disturbance of included subjects. Second, even within same subgroups of patients, the methodological approach for measuring gait speed varied within type of comparison. For example, comparisons of starting procedures (impact of a starting procedure with and without acceleration phase on resulting gait speed) in healthy patients were based on different testing components related to the length of the test distance, timing, or number of test repetitions, all factors potentially affecting resulting gait speed. Frequently, detailed description of underlying testing protocols was lacking suggesting that there were other unreported differences between studies. Furthermore, many studies published pooled data on mixed populations (e.g., young and old persons, patients with various types of disease) without reporting data of the subgroups. As a result, it was not possible to find sufficent numbers of comparable comparisons for subgroups of studies to permit pooling of results.

In the following paragraphs, we discuss our findings by individual testing procedure.

### Starting procedures

In the majority of reports, we found faster gait speed results for dynamic start protocols versus static start protocols. It is reasonable to conclude that a person needs time to accelerate before reaching usual gait speed. In a static start protocol, the acceleration phase is measured as part of the gait speed result, which can underestimate usual gait speed. In contrast, a dynamic start protocol removes the acceleration phase from the gait speed result and provides a more accurate measure of usual gait speed. Distances and times for acceleration and deceleration are of the upmost importance when measuring and comparing skills of atheletes. A study of elite female soccer players demonstrated that the mean distance to achieve a desired speed was 1.4 m for both accelerations (±0.15) and decelerations (±0.14) [42]. Further analysis showed that the mean and maximum distance per effort varied according to rate of acceleration and deceleration. In older people, starting procedures may have a clinically significant effect on gait speed results. The impact of static versus dynamic starting procedures may be even become more pronounced in patients with underlying diseases (e.g. an advanced Parkinson disease) that are marked by progressive decline in gait and muscle function. In these patients, additional time and distance will probably be needed to attain usual gait speed.

Based on these considerations, the acceleration phase (usually estimated at 1.5 to 2 m) should not be included in the measuremet of usual gait speed.

### Distance procedures

In our systematic review, overall median absolute gait speed results were faster for longer distances (ranging from 6.1 to 10 m) than for shorter distances (2.4 to 5 m). However, this difference might be minimal. In one of the included studies with low risk of bias, Bohannon et al. [19] found a statistically significant, but clinically irrelevant, difference of 0.01 m/sec between gait speed measured over 2.4 m and 6.1 m distances. The authors concluded that the shorter distance of 8 feet (= 2.4 m) was justifiable. From a clinical perspective, shorter distances are more feasible and preferrable. Thus, a distance of 2.4 to 3 m is probably sufficient to measure time of usual gait speed.

### Timing procedures

The median difference in gait speed results between automatic and manual timing protocols was zero, but some studies noted relevant differences. For example, Kim et al. [31] described a significantly slower gait speed result using manual timing compared to automatic timing. The authors argued that manual timing using a stopwatch might lead to misclassification of mobility status and suggested that automatic timing be used instead. A possible reason for this effect might be, that the handling of stopwatches is based on subjectivity, as assessors manually trigger the stopwatch too early after the starting line or stop the timing too late, suggesting that establishing clear criteria for stopping the test might reduce measurement variation of manual timing. On the basis of these reports, automatic timing seems to be preferrable as a standardized method of measuring gait speed.

### Surface procedures

Surface test procedures revealed the largest overall median effect on gait speed. Rough or soft surfaces resulted in significantly slower gait speed compared to hard walkway surfaces. These effects are plausible as it is a natural phenomenon that irregular surfaces result in a slowing of pace to avoid loss of balance or falling. Use of challenging surfaces are more appropriate for assessing balance and functional gait. If the goal of the test is to measure usual gait speed, a hard surface walkway would be the most appropriate and reliable choice.

### Walkway procedures

Effects for walkway procedures showed mixed results. We found reports of both faster and slower gait speed results for electronic walkways compared to usual walk tests. Peters [40] explained these variable effects based on the level of mobility status of individuals tested. While older people with mobility impairment had slower gait speed results on the electronic walkway, older people without relevant mobility impairment had faster gait speed results on the electronic walkway. Thus, the authors argued that people with mobility restriction may have perceived the mat of the electronic walkway GAITRite as a potential tripping hazard and therefore decreased their walking speed as a precautionary measure. This explanation would be analogous to the effect of hard versus soft (carpet) surfaces.

Moreover, underlying distances of the electronic walkway and the usual walk test could have biased the results of pairwise comparisons of walkways. While the electronic walkway GAITRite is a mat of 4.42 m, the usual walk test was variously defined among reports. For example, Peters [40] defined the usual walk test as a distance of 3 m. Thus, participants potentially had more distance to accelerate using the electronic walkway (4.42 m) and therefore potentially achieved a faster peak speed compared to the shorter usual walk test of 3 m.

Thus, electronic walkways do not reflect the patterns of usual gait speed, and results may vary according to type of electronic walkway.

### Finishing procedures

We did not find any study reporting on results of pairwise comparison of finishing procedures. We assume that in older patients, the effect of deceleration on gait speed could be similar to the effect seen with acceleration during starting procedures (i.e., as older patients may need distance to achieve their usual pace, they may also need distance for deceleration at the end of the test). Without taking this natural deceleration into account, the resulting gait speed may be underestimated. Therefore, similar to starting procedures, a distance of 1.5 to 2 m after the finishing line should be sufficient to account for deceleration.

## Limitations

There are several limitations to this study. First, statistical approaches differed substantially among the included studies. For example, most of the studies did not report an intracorrelation coefficient (e.g. ICC), thus limiting interpretation and pooling of results. Second, results have to be interpreted with caution, as risk of bias of included studies limits generalizibility of results. Third, while we used predefined selection criteria to select publications for this systematic search, it is possible that a publication was missed in the selection process. However, we limited selection bias by using reproducible predefined search strategies and two independent reviewers. We found sufficient basis to justify our conclusions. Fourth, we focussed our review on the most common testing procedures, but there are other methodological aspects that could affect gait speed. For example, the inclusion or exclusion of verbal instructions provided to patients on how to to walk “usually” could influence gait speed results. Also, the number of trial runs, use of an assistive device, or type of footwear could impact resulting gait speed. Finally, the quality of the design and reporting of the included studies was variable. Aspects not included in our quality measure might also have affected study results, such as asynchronous testing for comparing methods of testing.

## Implications

We believe that our results have several important research implications. To achieve generalizability and comparability across clinical trials that use gait speed as an outcome or a predictor, it is important to have a standardized testing protocol. Our review suggests that variation in testing procedures (e.g. different surfaces) can produce misleading results. Cut-off values for normal vs. abnormal gait speed results must be interpreted with caution, since these values are dependent on the particular testing protocols used to measure gait speed. Future research should focus on descriptive evaluation of gait speed testing using standardized methods to define valid cut-off values.

In summary, it is relevant to put our findings into context of clinical impact. We found mixed results for pairwise comparisons, partly exceeding and partly not exceeding MCID. The MCID of 0.1 m/sec was exceeded in selected pairwise comparisons on starting, distance, surface, walkway test procedures. Overall, we found for some testing procedures effects in both directions. For example, dynamic protocols resulting in faster gait speed results than static, but there was also the opposite effect showing higher gait speed results with a static compared with a dynamic protocol. Based on these results, one could argue, that both the dimension of exceeding MCID or not and the direction of the impact largely depend on the population tested. For example, while starting procedures might not be clinically significant in healthy, young particpants, it may exceed MCID in older people or patients. However, there is no evidence to predict, in what subpopulations testing procedures will relevantly affect resulting gait speed, thereby exceeding MCID. We did not identify a pairwise comparison of timing test procedures exceeding MCID, but several studies documented, that manual differed from automatic timing due to assessor performance.

In conclusion, this systematic review demonstrates that standardiziation of the test procedure for static versus dynamic start, length of test distance, surface of the walkway, and a valid method for timing are key for obtaining valid results that can be compared with norm values or be used for measuring change of gait performance in an individual over time. The findings of the present systematic review do not answer what the exact standards should be, but clearly demonstrate that standardization is essential. One example for standardizing gait speed measurement with available norm values is the dataset derived from the NIH toolbox [43]. By implementing standardized testing methods, clinicians and researchers will be better able to obtain reliable and valid measurement of gait speed.

## Supporting information

S1 Table Characteristics of pairwise comparisons of starting procedures (n = 14) (dynamic versus static start). (PDF)

S2 Table Characteristics of pairwise comparisons of distance test procedures (n = 14) (longer versus shorter distance). (PDF)

S3 Table Characteristics of pairwise comparisons of timing test procedures (n = 12) (automatic versus manual). (PDF)

S4 Table Characteristics of pairwise comparisons of surface test procedures (n = 6) (hard versus soft surface). (PDF)

S5 Table Characteristics of pairwise comparisons of walkway test procedures (n = 7) (electronic walkway versus usual walk test). (PDF)

S6 Table Impact of starting procedures on gait speed results (n = 14). (PDF)

S7 Table Impact of distances on gait speed results (n = 14). (PDF)

S8 Table Impact of timing procedures on the gait speed results (n = 12). (PDF)

S9 Table Impact of surfaces on gait speed results (n = 6). (PDF)

S10 Table Impact of walkway test procedures on gait speed results (n = 7). (PDF)

S11 Table Search strategies. (PDF)

S12 Table PRISMA checklist. (PDF)
