# Supplementary material for: Effect of testing procedures on gait speed measurement: A systematic review
Source: PLoS One. 2020 Jun 1;15(6):e0234200. doi: 10.1371/journal.pone.0234200 (PMC7263604; doi:10.1371/journal.pone.0234200)
Supplement: S1 Table — (PDF) [file pone.0234200.s001.pdf]

S1 Table. Characteristics of pairwise comparisons of starting procedures (n=14) (dynamic versus static start)

Author	Definition of dynamic start: Distance for acceleration	Subjects ≥60 years included	Subjects with disease included	Subjects using walking aid included	≥2 trial runs per test protocol	Test distance	Distance for deceleration (static/ dynamic)	Timing	Test surface
Amatachaya 2019a	2m	No	No	No	Yes (no data)[d]	10m	0m / 2m	Automatic	n.r.
Amatachaya 2019b	2m	Yes (no data)[a]	No	No	Yes (no data)[d]	10m	0m / 2m	Automatic	n.r.
Amatachaya 2019c	2m	Yes (no data)[a]	Yes (no data)[b]	No	Yes (no data)[d]	10m	0m / 2m	Automatic	n.r.
Johnson 2020a (4m distance)	3m	No	No	No	Yes (no data)[d]	4m	0m / 3m	Manual	Hard
Johnson 2020a (10m distance)	3m	No	No	No	Yes (no data)[d]	10m	0m / 3m	Manual	Hard
Johnson 2020b (4m distance)	3m	No	No	No	Yes (no data)[d]	4m	0m / 3m	Manual	Hard
Johnson 2020b (10m distance)	3m	No	No	No	Yes (no data)[d]	10m	0m / 3m	Manual	Hard
Kim (manual timer)	1.5m	Yes (no data)[a]	Yes (no data)[b]	No	Yes (with data)	4m	0m / 1.5m	Manual	Hard
Kim (automatic timer)	1.5m	Yes(no data) [a]	Yes (no data)[b]	No	Yes (with data)	4m	0m / 1.5m	Automatic	Hard
Lindholm	2m	Yes (no data)[a]	Yes (no data)[b]	Yes (no data)[c]	Yes (with data)	10m	0m / 0m	Manual	n.r.
Oh (manual timer)	2m	Yes (with data)	No	No	Yes (with data)	4m	0m / 2m	Manual	Hard
Oh (automatic timer)	2m	Yes (with data)	No	No	No	4m	0m / 2m	Automatic	Hard
Sustakoski	0.5m	Yes (no data)[a]	Yes (no data)[b]	No	No	4m	0m / 0.5m	Manual	Hard
Warden	2m	Yes (no data)[a]	Yes (no data)[b]	Yes (no data)[c]	Yes (with data)	4m	0m / 2m	Automatic	n.r.

n.r.: not reported

[a] Results for subgroup of persons aged ≥60 years were not reported

[b] Results for subgroup of persons with disease were not reported

[c] Results for subgroup of persons using a walking aid were not reported

[d] Results were reported as the mean value of all trial runs, results were not reported for each trial run separately
