# Supplementary material for: Effect of testing procedures on gait speed measurement: A systematic review
Source: PLoS One. 2020 Jun 1;15(6):e0234200. doi: 10.1371/journal.pone.0234200 (PMC7263604; doi:10.1371/journal.pone.0234200)
Supplement: S2 Table — (PDF) [file pone.0234200.s002.pdf]

S2 Table. Characteristics of pairwise comparisons of distance test procedures (n=14) (longer versus shorter distance)

Author	Definition of test distance (short/ long)	Subjects ≥60 years included	Subjects with disease included	Subjects using walking aid included	≥2 trial runs per test protocol	Distance for acceleration (short/long)	Distance for deceleration (short/long)	Timing	Test surface
Amatachaya 2019a	4m/ 10m	No	No	No	Yes (no data)[d]	3m/ 2m	3m/ 2m	Automatic	n.r.
Amatachaya 2019b	4m/ 10m	Yes (no data)[a]	No	No	Yes (no data)[d]	3m/ 2m	3m/ 2m	Automatic	n.r.
Amatachaya 2019c	4m/ 10m	Yes (no data)[a]	Yes (no data)[b]	No	Yes (no data)[d]	3m/ 2m	3m/ 2m	Automatic	n.r.
Bohannon	2.4m/ 6.1m	Yes (with data)	Yes (no data)[b]	No	No	0m/ 0m	0m/ 0m	Manual	n.r.
Johnson 2020a (static start)	4m/ 10m	No	No	No	Yes (no data)[d]	0m/ 0m	0m/ 0m	Manual	Hard
Johnson 2020a (dynamic start)	4m/ 10m	No	No	No	Yes (no data)[d]	3m/ 3m	3m/ 3m	Manual	Hard
Johnson 2020b (static start)	4m/ 10m	No	No	No	Yes (no data)[d]	0m/ 0m	0m/ 0m	Manual	Hard
Johnson 2020b (dynamic start)	4m/ 10m	No	No	No	Yes (no data)[d]	3m/ 3m	3m/ 3m	Manual	Hard
Karpman	4m/ 10m	Yes (no data)[a]	Yes (no data)[b]	Yes (no data)[c]	Yes (no data)[d]	2m/ 2m	2m/ 2m	Automatic	Hard
Lyons	3m/ 6m	Yes (no data)[a]	Yes (no data)[b]	n.r.	Yes (with data)	0m/ 0m	0.5m/ 0.5m	Manual	n.r.
Ng 2012	5m/ 10m	Yes (no data)[a]	Yes (no data)[b]	Yes (no data)[c]	Yes (no data)[d]	2m/ 2m	2m/ 2m	Manual	n.r.
Ng 2013	5m/ 10m	Yes (no data)[a]	No	No	No	2m/ 2m	2m/ 2m	Manual	n.r.
Pasma	4m/ 10m	Yes (with data)	Yes (no data)[b]	Yes (no data)[c]	Yes (no data)[d]	0m/ 2.5m	1m/ 2.5m	Manual	n.r.
Peters 2013	4m/ 10m	Yes (with data)	Yes (no data)[b]	Yes (no data)[c]	Yes (with data)	2m/ 5m	2m/ 2m	Manual	n.r.

n.r.: not reported

[a] Results for subgroup of persons aged ≥60 years were not reported

[b] Results for subgroup of persons with disease were not reported

[c] Results for subgroup of persons using a walking aid were not reported

[d] Results were reported as the mean value of all trial runs, results were not reported for each trial run separately
