# Supplementary material for: Effect of testing procedures on gait speed measurement: A systematic review
Source: PLoS One. 2020 Jun 1;15(6):e0234200. doi: 10.1371/journal.pone.0234200 (PMC7263604; doi:10.1371/journal.pone.0234200)
Supplement: S3 Table — (PDF) [file pone.0234200.s003.pdf]

S3 Table. Characteristics of pairwise comparisons of timing test procedures (n=12) (automatic versus manual)

Author	Subjects ≥60 years included	Subjects with disease included	Subjects using walking aid included	≥2 trial runs per test protocol	Distance for acceleration	Test distance	Distance for deceleration	Test surface
Barry	Yes (with data)	Yes (no data)[b]	Yes (no data)[c]	Yes (no data)[d]	0.6m	4.27m	0.9m	Hard
Bisca (walkway distance 4m)	Yes (with data)	Yes (with data)	Not specified	Yes (no data)	0m	4m	0m	Hard
Bisca (walkway distance 8m)	Yes (with data)	Yes (with data)	n.r.	Yes (no data)	2m	4m	2m	Hard
Karpman	Yes (no data)[a]	Yes (with data)	Yes (no data)[c]	Yes (no data)[d]	2m	4m	2m	Hard
Kim (static start)	Yes (with data)	Yes (no data)[b]	No	Yes (with data)	0m	4m	0m	Hard
Kim (dynamic start)	Yes (with data)	Yes (no data)[b]	No	Yes (with data)	1.5m	4m	1.5m	Hard
Oh (static start)	Yes (with data)	No	No	Yes (with data)	0m	4m	0m	Hard
Oh (dynamic start)	Yes (with data)	No	No	Yes (with data)	2m	4m	2m	Hard
Peters 2013 (4m test distance)	Yes (with data)	Yes (no data)[b]	Yes (no data)[c]	Yes (with data)	2m	4m	2m	n.r.
Peters 2013 (10m test distance)	Yes (with data)	Yes (no data)[b]	Yes (no data)[c]	Yes (with data)	5m	10m	5m	n.r.
Sustakoski	Yes (with data)	Yes (no data)[b]	n.r.	No	0.6m	4m	0.6m	Hard
Warden	Yes (no data)[a]	Yes (no data)[b]	Yes (no data)[c]	Yes (with data)	0m	4m	0m	n.r.

n.r.: not reported

[a] Results for subgroup of persons aged ≥60 years were not reported

[b] Results for subgroup of persons with disease were not reported

[c] Results for subgroup of persons using a walking aid were not reported

[d] Results were reported as the mean value of all trial runs, results were not reported for each trial run separately
