# Supplementary material for: Effect of testing procedures on gait speed measurement: A systematic review
Source: PLoS One. 2020 Jun 1;15(6):e0234200. doi: 10.1371/journal.pone.0234200 (PMC7263604; doi:10.1371/journal.pone.0234200)
Supplement: S4 Table — (PDF) [file pone.0234200.s004.pdf]

S4 Table. Characteristics of pairwise comparisons of surface test procedures (n=6) (hard versus soft surface)

Author	Subjects ≥60 years included	Subjects with disease included	Subjects using walking aid included	≥2 trial runs per test protocol	Distance for acceleration	Test distance	Distance for deceleration	Timing
Promkeaw 2019a	Yes (no data)[a]	Yes (with data)	Yes (no data)[b]	No	3m	4m	3m	n.r.
Promkeaw 2019b	Yes (no data)[a]	No	No	No	3m	4m	3m	n.r.
Promkeaw 2019a	Yes (no data)[a]	Yes (with data)	Yes (no data)[b]	No	3m	4m	3m	n.r.
Promkeaw 2019b	Yes (no data)[a]	No	No	No	3m	4m	3m	n.r.
Stephens	Yes (no data)[a]	Yes (with data)	n.r.	Yes (with data)	2m	6m	2m	n.r.
Willmott	Yes (with data)	Yes (with data)	n.r.	No	2.25m	10m	2.25m	n.r.

n.r.: not reported

[a] Results for subgroup of persons aged ≥60 years were not reported

[b] Results for subgroup of persons using a walking aid were not reported
