# Supplementary material for: Effect of testing procedures on gait speed measurement: A systematic review
Source: PLoS One. 2020 Jun 1;15(6):e0234200. doi: 10.1371/journal.pone.0234200 (PMC7263604; doi:10.1371/journal.pone.0234200)
Supplement: S5 Table — (PDF) [file pone.0234200.s005.pdf]

S5 Table. Characteristics of pairwise comparisons of walkway test procedures (n=7) (electronic walkway versus usual walk test)

Author	Subjects ≥60 years included	Subjects with disease included	Subjects using walking aid included	≥2 trial runs per test protocol	Distance for acceleration (usual/ electronic	Test Distance (usual/ electronic	Distance for deceleration (usual/ electronic)	Timing
Bryant 2013	Yes (with data)	Yes (with data)	No	No	0.6m/0.6m	5m/5m	0.6m/0.6m	Automatic
Bryant 2015	Yes (with data)	Yes (with data)	n.r.	Yes (no data)[d]	0m/0m	5m/4.27m	0m/0m	Automatic
Cleland	Yes (no data)[a]	Yes (with data)	Yes (no data)[c]	Yes (no data)[d]	2m/2m	5m/4.27m	2m/2m	Automatic
Peters 2014a	Yes (no data)[a]	Yes(with data)	Yes (no data)[c]	Yes (with data)	2m/2m	3m/4.42m	2m/2m	Automatic
Peters 2014b	Yes (no data)[a]	Yes (with data)	Yes (no data)[c]	Yes (with data)	2m/2m	3m/4.42m	2m/2m	Automatic
Peters 2014c	Yes (no data)[a]	Yes (with data)	Yes (no data)[c]	Yes (with data)	2m/2m	3m/4.42m	2m/2m	Automatic
Sustakoski	Yes (with data)	Yes (no data)[b]	n.r.	No	0.6m/2m	4m/4m	0.6m/2m	Automatic

n.r.: not reported

[a] Results for subgroup of persons aged ≥60 years were not reported

[b] Results for subgroup of persons with disease were not reported

[c] Results for subgroup of persons using a walking aid were not reported

[d] Results were reported as the mean value of all trial runs, results were not reported for each trial run separately
