# Supplementary material for: Effect of testing procedures on gait speed measurement: A systematic review
Source: PLoS One. 2020 Jun 1;15(6):e0234200. doi: 10.1371/journal.pone.0234200 (PMC7263604; doi:10.1371/journal.pone.0234200)
Supplement: S6 Table — (PDF) [file pone.0234200.s006.pdf]

S6 Table. Impact of starting procedures on gait speed results (n=14)

Author	Gait speed static start: (m/sec) mean (SD)	Gait speed dynamic start: (m/sec) mean (SD)	Mean difference gait speed dynamic vs. static start: (m/sec) (95%CI)	p-value[a)]	Intraclass correlation coefficient) (95% CI)	Risk of bias (%)
Amatachaya 2019a	1.27 (1.69)	1.38 (0.18)	0.11 (n.r.)	<0.001	n.r.	87.5
Amatachaya 2019b	1.13 (0.14)	1.18 (0.16)	0.05 (n.r.)	0.018	n.r.	87.5
Amatachaya 2019c	0.53 (0.17)	0.59 (0.20)	0.06 (n.r.)	0.004	n.r.	87.5
Johnson 2020a (4m distance)	1.05 (0.03)	1.4 (0.04)	0.35 (n.r.)	<0.001	n.r.	66.7
Johnson 2020a (10m distance)	1.28 (0.03)	1.44 (0.03)	0.16 (n.r.)	<0.001	n.r.	66.7
Johnson 2020b (4m distance)	1.34 (0.03)	1.41 (0.03)	0.07 (n.r.)	0.075	n.r.	66.7
Johnson 2020b (10m distance)	1.44 (0.03)	1.45 (0.03)	0.01 (n.r.)	1.00	n.r.	66.7
Kim (manual timer)	1.06 (0.20)	1.12 (0.22)	0.06 (n.r.)	<0.001	R^2 = 0.76;	33.3
Kim (automatic timer)	1.13 (0.24)	1.19 (0.25)	0.06 (n.r.)	<0.001	R^2=0.72	33.3
Lindholm	1.11 (0.29)	1.09 (0.28)	-0.02 (-0.008 to -0.026)	<0.001	0.98 (0.97 to 0.99)	36.3
Oh (manual timer)	1.21 (0.21)	1.27 (0.20)	0.06 (0.03 to 0.08)	<0.001	n.r.	66.7
Oh (automatic timer)	1.20 (0.22)	1.22 (0.21)	0.02 (0.002 to 0.05)	0.135	n.r.	66.7
Sustakoski	0.97 (0.23)	1.13 (0.25)	0.16 (0.14 to 0.19)	<0.001	n.r.	55.6
Warden	n.r.	n.r.	0.05 (-0.15 to 0.24)	>0.05	n.r.	55.6

Abbreviations: SD, standard deviation; CI, confidence interval; n.r., not reported. For characteristics of studies, see Table 1. For definition of risk of bias, see Methods section.

a) p-value reported for comparisons of means method 1 vs. 2
