# Supplementary material for: Effect of testing procedures on gait speed measurement: A systematic review
Source: PLoS One. 2020 Jun 1;15(6):e0234200. doi: 10.1371/journal.pone.0234200 (PMC7263604; doi:10.1371/journal.pone.0234200)
Supplement: S7 Table — (PDF) [file pone.0234200.s007.pdf]

**S7 Table. Impact of distances on gait speed results (n=14)**

| Author | Length of tested distances | Gait speed of shorter distance: (m/sec) mean (SD) | Gait speed of longer distance: (m/sec) mean (SD) | Mean difference gait speed longer vs. shorter distance: (m/sec) (95%CI) | p-value[a)] | Intraclass correlation coefficient) (95% CI) | Risk of bias (%) |
|---|---|---|---|---|---|---|---|
| Amatachaya 2019a | 4m vs. 10m | 1.34 (0.23) | 1.37 (0.18) | 0.03 (n.r.) | 0.212 | n.r. | 87.5 |
| Amatachaya 2019b | 4m vs. 10m | 1.22 (0.14) | 1.17 (0.17) | -0.05 (n.r.) | 0.210 | n.r. | 87.5 |
| Amatachaya 2019c | 4m vs. 10m | 0.56 (0.19) | 0.59 (0.20) | 0.03 (n.r.) | 0.054 | n.r. | 87.5 |
| Bohannon | 8 feet (2.4m) vs. 20 feet (6.1m) | 0.99 (0.26) | 1.00 (0.24) | 0.01 (n.r.) | 0.002 | r=0.933, p<0.001 | 20.0 |
| Johnson 2020a (static start) | 4m vs. 10m | 1.05 (0.03) | 1.28 (0.03) | 0.23 (n.r.) | <0.001 | n.r. | 66.7 |
| Johnson 2020a (dynamic start) | 4m vs. 10m. | 1.4 (0.0)4 | 1.44 (0.03) | 0.04 (n.r.) | 0.14 | n.r. | 66.7 |
| Johnson 2020b (static start) | 4m vs. 10m | 1.34 (0.03) | 1.44 (0.03) | 0.1 (n.r.) | <0.001 | n.r. | 66.7 |
| Johnson 2020b (dynamic start) | 4m vs. 10m | 1.41 (0.03) | 1.45 (0.03) | 0.04 (n.r.) | 0.007 | n.r. | 66.7 |
| Karpman | 4m vs. 10m | 1.14 (0.24) | 1.27 (0.24) | 0.13 (-0.07 to 0.31)[c)] | <0.001[c)] | n.r. | 66.7 |
| Lyons | 3m vs. 6m | 0.81 (0.23) | 0.94 (0.22) | 0.13 (n.r.) | n.r. | 0.80 (0.73 to 0.75) | 33.3 |
| Ng 2012 | 5m vs. 10m | 0.76 (0.26) | 0.79 (0.27) | 0.03 (n.r.) | 0.093[a)] | n.r. | 66.7 |
| Ng 2013 | 5m vs. 10m | 1.22 (0.18) | 1.19 (0.20) | -0.03 (n.r.) | 0.319[b)] | n.r. | 77.8 |
| Pasma | 4m vs. 10m | 0.72 (0.27) | 0.84 (0.28) | 0.12 (0.10 to 0.13) | <0.001 | 0.79 (0.44 to 0.90) | 50.0 |
| Peters 2013 | 4m vs. 10m | 0.97 (0.22) | 0.97 (0.22) | 0.0007 (-0.17 to 0.17) | 0.957 | 0.93 (0.87 to 0.96) | 30.0 |

*Abbreviations: SD, standard deviation; CI, confidence interval; n.r., not reported. For characteristics of studies, see Table 1. For definition of risk of bias, see Methods section.*

a) p-value reported for comparisons of means method 1 vs. 2
b) p-value stated on an overall difference between three testing protocols (5m vs. 8m vs. 10m)
c) Reported 95% Confidence interval from Bland-Altman analysis. However, reported p-value is from a comparison of means.
