# Supplementary material for: Effect of testing procedures on gait speed measurement: A systematic review
Source: PLoS One. 2020 Jun 1;15(6):e0234200. doi: 10.1371/journal.pone.0234200 (PMC7263604; doi:10.1371/journal.pone.0234200)
Supplement: S8 Table — (PDF) [file pone.0234200.s008.pdf]

S8 Table. Impact of timing procedures on the gait speed results (n=12)

Author	Gait speed of manual timing: (m/sec) mean (SD)	Gait speed of automatic timing: (m/sec) mean (SD)	Mean difference gait speed automatic vs. manual timing: (m/sec) (95%CI)	p-value[a)]	Intraclass correlation coefficient) (95% CI)	Risk of bias (%)
Barry	0.80 (0.25)	0.80 (0.25)	-0.003 (0.03 (SD))	0.43	n.r.	22.2
Bisca (walkway distance 4m)	1.07 (0.24)	1.05 (0.21)	-0.02 (n.r.)	0.17	0.91 (0.84 to 0.95)	50.0
Bisca (walkway distance 8m)	1.32 (1.10 to 1.40) Median (IQR)	1.31 (1.20 to 1.50) Median (IQR)	-0.01 (n.r.)	0.12	0.95 (0.92 to 0.97)	50.0
Karpman	1.13 (0.23)	1.14 (0.24)	0.01 (-0.1 to 0.08)[b)]	n.r.	0.99 (0.98 to 0.99)	66.7
Kim (static start protocol)	1.06 (0.20)	1.13 (0.24)	0.07 (0.06 to 0.08)	<0.001	R^2= 0.78	33.3
Kim (dynamic start protocol)	1.12 (0.22)	1.19 (0.25)	0.07 (0.06 to 0.08)	<0.001	R^2= 0.81	33.3
Oh (using static start)	1.21 (0.21)	1.20 (0.22)	-0.01 (-0.04 to 0.10)	0.255	n.r.	66.7
Oh (using dynamic start)	1.27 (0.20)	1.22 (0.21)	-0.05 (-0.06 to -0.03)	<0.001	n.r.	66.7
Peters 2013 (4m test distance)	0.97 (0.22)	0.97 (0.22)	0.00 (-0.05 to 0.05)	n.r.	0.99 (0.988 to 0.996)	30.0
Peters 2013 (10m test distance)	0.97 (0.22)	0.97 (0.22)	0.00 (-0.02 to 0.02)	n.r.	1.00 (0.999 to 1.00)	30.0
Sustakoski	1.13 (0.25)	1.14 (0.25)	0.01 (-0.02 to 0.03)	0.717	n.r.	55.6
Warden	n.r.	n.r.	0.01 (-0.14 to 0.16)	>0.05	n.r.	55.6

Abbreviations: SD, standard deviation; CI, confidence interval; n.r., not reported. For characteristics of studies, see Table 1. For definition of risk of bias, see Methods section.

a) p-value reported for comparisons of means method 1 vs. 2
b) 95% Confidence interval reported from Bland-Altman analysis
