# Supplementary material for: Effect of testing procedures on gait speed measurement: A systematic review
Source: PLoS One. 2020 Jun 1;15(6):e0234200. doi: 10.1371/journal.pone.0234200 (PMC7263604; doi:10.1371/journal.pone.0234200)
Supplement: S9 Table — (PDF) [file pone.0234200.s009.pdf]

S9 Table. Impact of surfaces on gait speed results (n=6)

Author	Description of hard surface	Description of soft surface	Gait speed of hard surface: (m/sec) mean (SD)	Gait speed of soft surface: (m/sec) mean (SD)	Mean difference gait speed testing soft vs. hard surface: (m/sec) (95%CI)	p-value[a)]	Intraclass correlation coefficient) (95% CI)	Risk of bias (%)
Promkeaw 2019a	Hard	Artificial Grass	0.66 (0.41 to 0.99) Median (IQR)	0.55 (0.33 to 0.68) Median (IQR)	-0.11 (n.r.)	<0.001	n.r.	75.0
Promkeaw 2019b	Hard	Artificial Grass	1.10 (0.17 to 1.10) Median (IQR)	1.00 (0.88-1.10) Median (IQR)	-0.10 (n.r.)	<0.001	n.r.	75.0
Promkeaw 2019a	Hard	Soft	0.66 (0.41 to 0.99) Median (IQR)	0.48 (0.31 to 0.60) Median (IQR)	-0.18 (n.r.)	<0.001	n.r.	75.0
Promkeaw 2019b	Hard	Soft	1.10 (0.17 to 1.10) Median (IQR)	0.92 (0.82 to 0.92) Median (IQR)	-0.18 (n.r.)	<0.001	n.r.	75.0
Stephens	Parquetry	Carpet	0.79 (0.23)	0.75 (0.24)	-0.04 (n.r.)	<0.05	r=0.92	44.4
Willmott	Vinyl	Carpet	0.40 (0.17)	0.48 (0.19)	0.08 (n.r.)	<0.001[b)]	N.r.	88.9

Abbreviations: SD, standard deviation; CI, confidence interval; n.r., not reported; IQR, interquartile range. For characteristics of studies, see Table 1. For definition of risk of bias, see Methods section.

a) p-value reported for comparisons of means method 1 vs. 2
b) One-tailed p-value indicated
