# Supplementary material for: Effect of testing procedures on gait speed measurement: A systematic review
Source: PLoS One. 2020 Jun 1;15(6):e0234200. doi: 10.1371/journal.pone.0234200 (PMC7263604; doi:10.1371/journal.pone.0234200)
Supplement: S10 Table — (PDF) [file pone.0234200.s010.pdf]

S10 Table. Impact of walkway test procedures on gait speed results (n=7)

Author	Description of usual walk test	Description of electronic walkway	Gait speed of usual walk test: (m/sec) mean (SD)	Gait speed of electronic walkway: (m/sec) mean (SD)	Mean difference gait speed testing usual electronic walkway vs. usual walk test: (m/sec) (95%CI)	p-value[a)]	Intraclass correlation coefficient) (95% CI)	Risk of bias (%)
Bryant 2013	Floor (5m)	Walkway GAITRite	0.82 (0.24)	0.86 (0.25)	0.04 (n.r.)	0.018	0.96 (0.92 to 0.98)	77.8
Bryant 2015	Overground (5m)	Walkway GAITRite	0.97 (0.29)	1.10 (0.32)	0.04 (n.r.)	0.016	0.85 (0.58 to 0.95)	100.0
Cleland	10MWT	Walkway GAITRite	0.75 (0.21)	0.67 (0.16)	-0.08 (-0.05 to -0.10)	<0.001	0.77 (0.46 to 0.89)	88.9
Peters 2014a	Floor (3MWT)	Walkway GAITRite	0.89 (0.15)	1.03 (0.16)	0.14 (n.r.)	<0.05	0.49 (-0.09 to 0.78)	44.4
Peters 2014b	Floor (3MWT)	Walkway GAITRite	0.52 (0.10)	0.56 (0.11)	0.04 (n.r.)	<0.05	0.75 (0.22 to 0.90)	44.4
Peters 2014c	Floor (3MWT)	Walkway GAITRite	0.27 (0.11)	0.25 (0.11)	-0.02 (n.r.)	<0.05	0.89 (0.72 to 0.95)	44.4
Sustakoski	Overground (4m)	Walkway GaitMat II	1.14 (0.25)	1.06 (0.26)	0.07 (0.04-0.10)	<0.001	n.r.	55.6

Abbreviations: SD, standard deviation; n.r., not reported; CI, confidence interval; 10MWT, 10-m walk test; 3MWT, 3-m walk test. For characteristics of studies, see Table 1. For definition of risk of bias, see Methods section.

a) p-value reported for comparisons of means method 1 vs. 2
