# Supplementary material for: Effect of testing procedures on gait speed measurement: A systematic review
Source: PLoS One. 2020 Jun 1;15(6):e0234200. doi: 10.1371/journal.pone.0234200 (PMC7263604; doi:10.1371/journal.pone.0234200)
Supplement: S11 Table — (PDF) [file pone.0234200.s011.pdf]

S11 Table. Search strategies

**Search Strategy No. 1 Pubmed (#1):**
(walk test) AND (gait speed) NOT review[Publication Type] NOT minute [title]
**Search Strategy No. 2 Pubmed (#2):**
(gait speed) AND (testing protocol) NOT "review"[Publication Type]
**Search Strategy No. 3 Embase (#3):**
'walk test' AND 'gait speed' AND [article]/lim AND [english]/lim AND [embase]/lim
**Search Strategy No. 4 Embase (#4):**
('gait'/exp OR gait) AND ('speed'/exp OR speed) AND ('testing'/exp OR testing) AND ('protocol'/exp OR protocol) AND [article]/lim AND [english]/lim AND [humans]/lim AND [clinical study]/lim AND [embase]/lim
